# Adjunctive *Acorus tatarinowii* essential-oil ear-canal packing for idiopathic sudden sensorineural hearing loss: study protocol for a single-center, randomized, double-blind, placebo-controlled clinical trial

**DOI:** 10.3389/fphar.2026.1875920

**Published:** 2026-09-30

**Authors:** Sibei Chen, Handong Xu, Xiao He, Xuxia Tang

**Affiliations:** 1 The First Affiliated Hospital of Zhejiang Chinese Medical University (Zhejiang Provincial Hospital of Chinese Medicine), Hangzhou, China; 2 The First Clinical Medical College, Zhejiang Chinese Medical University, Hangzhou, China

**Keywords:** *Acorus tatarinowii* Schott, adjunctive therapy, double-blind trial, essential oil, idiopathic sudden sensorineural hearing loss, randomized controlled trial

## Abstract

**Background:**

Idiopathic sudden sensorineural hearing loss (ISSNHL) is an acute otologic emergency, and hearing recovery remains highly variable and frequently unsatisfactory. *Acorus tatarinowii* Schott has long been used in traditional Chinese medicine to treat hearing impairment and related auditory disorders. Its essential oil (SCP-oil) has demonstrated anti-inflammatory, antioxidant, and neuroprotective activities in preclinical studies. Advances in local otologic drug-delivery strategies suggest that administration through the external auditory canal may offer a practical, noninvasive approach to local treatment. This study therefore aims to evaluate the efficacy and safety of adjunctive SCP-oil ear-canal packing in patients with ISSNHL.

**Methods and analysis:**

This is a single-center, randomized, double-blind, placebo-controlled clinical trial to be conducted at Zhejiang Provincial Hospital of Chinese Medicine. The trial has been registered with the International Traditional Medicine Clinical Trial Registry (ITMCTR2026000919). A total of 258 eligible participants will be randomly assigned in a 1:1 ratio to receive conventional therapy combined with either SCP-oil ear-canal packing or glycerin placebo ear-canal packing for 14 consecutive days. The primary outcome is the study-defined total markedly effective rate of hearing recovery at week 2, defined as the proportion of participants achieving complete recovery or marked improvement, as assessed by pure-tone audiometry. Secondary outcomes include the mean improvement in hearing thresholds across the affected frequencies, the hearing-recovery categories, tinnitus severity measured using the Visual Analog Scale (VAS), depressive symptoms assessed using the Self-Rating Depression Scale (SDS), and sleep quality assessed using the Pittsburgh Sleep Quality Index (PSQI). Efficacy outcomes will be assessed at baseline and week 2, with an additional safety assessment conducted at week 4. Adverse events will be monitored throughout the study.

**Discussion:**

This trial will provide evidence on the efficacy, safety, and tolerability of adjunctive SCP-oil ear-canal packing for ISSNHL, including its effects on hearing recovery and patient-reported outcomes. The findings may also inform the design of future multicenter clinical trials.

## 1 Introduction

Idiopathic sudden sensorineural hearing loss (ISSNHL) is characterized by the rapid onset of sensorineural hearing loss without an identifiable cause after routine clinical evaluation. Population-based epidemiologic studies have reported an increasing incidence of sudden sensorineural hearing loss in recent years. Patients with ISSNHL may present with varying degrees of hearing impairment, which is typically unilateral and may be accompanied by tinnitus, aural fullness, vertigo, dizziness, or imbalance. Its pathophysiology remains incompletely understood but is thought to involve multiple mechanisms, including microvascular compromise, viral infection or inflammatory injury, immune-mediated responses, and oxidative stress. In addition to auditory impairment, ISSNHL can adversely affect speech communication, sleep, occupational functioning, and social participation and may be associated with anxiety, depressive symptoms, and reduced quality of life (Lee and Chung, 2024; Chandrasekhar et al., 2019; Kim et al., 2018; Stachler et al., 2012). Despite available therapies, hearing recovery remains highly variable, and a substantial proportion of patients do not achieve satisfactory recovery. The uncertain etiology and heterogeneous clinical course of ISSNHL further complicate treatment selection and the interpretation of therapeutic effects. Therefore, the development and evaluation of additional therapeutic strategies for ISSNHL are warranted (Lee and Chung, 2024; Chandrasekhar et al., 2019; Rodríguez-Izquierdo et al., 2025).

*Acorus tatarinowii* Schott has long been used in traditional Chinese medicine, particularly for auditory and neurological disorders (Castañeda et al., 2019). Its dried rhizome is used medicinally, and the essential oil is considered one of its major bioactive fractions (Wang et al., 2023; Wen et al., 2023). Preclinical studies have reported anti-inflammatory, antioxidant, and neuroprotective effects of SCP-oil and its major volatile constituents, including α-, β-asarone, and eugenol (Wen et al., 2023; Balakrishnan et al., 2022; Xu et al., 2023). These findings provide a pharmacological rationale for evaluating SCP-oil as an adjunctive treatment for ISSNHL.

From a drug-delivery perspective, current treatment approaches for ISSNHL mainly include systemic pharmacotherapy and intratympanic administration. Systemic therapy may be limited by variable therapeutic responses and the risk of systemic adverse effects, whereas intratympanic administration is invasive and may cause procedure-related discomfort or complications (Lee and Chung, 2024; Chandrasekhar et al., 2019; Rodríguez-Izquierdo et al., 2025; Joshua et al., 2022; Ramzi et al., 2025). In this context, noninvasive local delivery through the external auditory canal may represent a clinically feasible adjunctive approach that avoids tympanic membrane puncture while permitting drug administration to the affected ear (Zhang et al., 2021; Xu et al., 2024; Cheng et al., 2025; Patel et al., 2019). External auditory canal administration has also been described in traditional medical practice for ear disorders. For example, historical records in *The Handbook of Prescriptions for Emergencies* describe the use of *A. tatarinowii* with cotton placed in the external auditory canal for deafness, which provides a historical basis for the present intervention. In addition, modern otologic drug-delivery studies suggest that selected formulations applied to the external auditory canal may achieve transtympanic permeation under appropriate conditions (Yang et al., 2016). Although whether and by what route externally administered SCP-oil reaches the inner ear remain unclear preliminary animal experiments conducted by our group have provided qualitative evidence of possible cochlear exposure, supporting the biological plausibility of this administration route. Nevertheless, the precise transport pathway, pharmacokinetic profile, extent of cochlear exposure, and whether therapeutically meaningful cochlear concentrations can be achieved remain to be determined. Taken together, the historical context and preliminary experimental observations provide a rationale for evaluating SCP-oil ear-canal packing as a potential noninvasive local adjunct to conventional therapy for ISSNHL.

Accordingly, we developed a standardized protocol for SCP-oil ear-canal packing and designed this clinical trial at Zhejiang Provincial Hospital of Chinese Medicine to evaluate the efficacy and safety of this adjunctive approach in patients with ISSNHL. This trial aims to provide preliminary clinical evidence regarding the efficacy, safety, and tolerability of SCP-oil ear-canal packing as an adjunct to conventional therapy for ISSNHL.

## 2 Methods and analysis

### 2.1 Study design

This single-center, randomized, double-blind, placebo-controlled clinical trial will be conducted at Zhejiang Provincial Hospital of Chinese Medicine. A total of 258 eligible hospitalized patients with ISSNHL will be enrolled and randomly assigned in a 1:1 ratio to receive conventional therapy combined with either SCP-oil ear-canal packing or glycerin placebo ear-canal packing. The intervention will last 14 consecutive days, and a safety follow-up will be conducted at week 4. Outcome assessments will be conducted at baseline and week 2 and will include pure-tone audiometry, the tinnitus Visual Analog Scale (VAS), the Self-Rating Depression Scale (SDS), and the Pittsburgh Sleep Quality Index (PSQI).

This trial has been registered in the International Traditional Medicine Clinical Trial Registry (ITMCTR2026000919). This protocol was developed and is reported in accordance with the SPIRIT guidelines. The study flow diagram is presented in Figure 1, and the study schedule is provided in Table 1.

**FIGURE 1:**
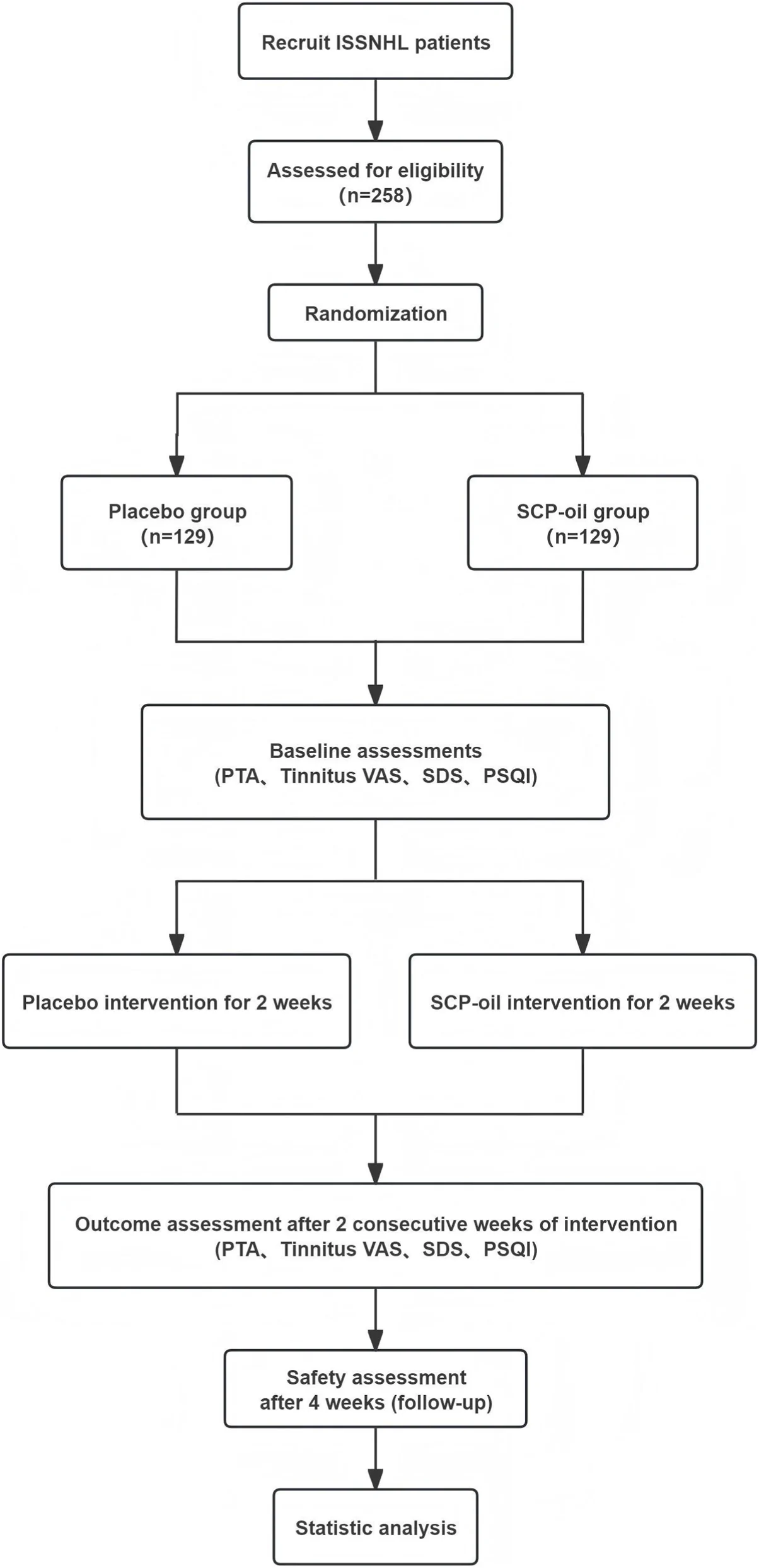
Trial flow diagram.

**TABLE 1:** Trial schedule.

Study procedure	Baseline	Treatment phase		Follow-up
	Week 0	Week 1	Week 2	Week 4
ISSNHL participants				
Eligibility assessment	√	​	​	​
Informed consent	√	​	​	​
Baseline assessment	√	​	​	​
Intervention	​	√	√	​
Outcome measures				
PTA	√	​	√	​
Tinnitus VAS	√	​	√	​
SDS	√	​	√	​
PSQI	√	​	√	​
Safety assessment	√	√	√	√

### 2.2 Inclusion criteria

Eligibility will be determined in accordance with the *Guideline for Diagnosis and Treatment of Sudden Sensorineural Hearing Loss (2026 Practical Edition)*, issued by the Chinese Society of Otorhinolaryngology Head and Neck Surgery of the Chinese Medical Association.

Participants who meet all of the following criteria will be eligible for inclusion:

1. Unilateral sudden-onset sensorineural hearing loss occurring within 72 h, with a decrease of ≥20 dB HL at ≥2 adjacent frequencies (Editorial Board of Chinese Journal of Otorhinolaryngology Head and Neck Surgery, Society of Otorhinolaryngology Head and Neck Surgery, Chinese Medical Association, National Clinical Research Center for Otorhinolaryngologic Diseases, 2026);
2. No definite etiology identified after routine clinical evaluation, with systemic and local causes excluded;
3. Age between 18 and 70 years, with no sex restrictions;
4. No severe hepatic, renal, or other systemic dysfunction that would preclude standard treatment or study participation;
5. Provision of written informed consent.

### 2.3 Exclusion criteria

Participants who meet any of the following criteria will be excluded:

1. A pure-tone average (PTA) > 90 dB HL in the unaffected ear;
2. Pregnancy or lactation;
3. Inability to complete follow-up or outcome assessment;
4. Known allergy or suspected hypersensitivity to any medications used in the study;
5. Evidence of alternative causes of hearing loss, including middle-ear pathology, vestibular schwannoma, or functional hearing loss, also known as pseudohypacusis.

### 2.4 Recruitment process

Potential participants will be identified among hospitalized patients admitted to the Department of Otolaryngology at Zhejiang Provincial Hospital of Chinese Medicine. Trained research assistants will assist with the identification of potential participants, preliminary eligibility screening, and completion of screening documentation under the supervision of study physicians. Final eligibility assessment, confirmation of the diagnosis of idiopathic sudden sensorineural hearing loss, and determination of medical suitability for trial participation will be performed by licensed otolaryngologists or qualified study physicians. All physicians and research assistants involved in recruitment, screening, informed consent, allergy testing, and trial documentation will receive protocol-specific training before recruitment begins. Written informed consent will be obtained before any study-specific procedure, including allergy screening, is performed.

After written informed consent has been obtained and before randomization, all potential participants will undergo a skin allergy screening test for both study preparations. The screening procedure is illustrated in Figure 2. Both study preparations will be applied separately to two distinct postauricular skin sites, with each application covering an area approximately 1 cm in diameter. After 15 min, both sites will be examined by a trained study physician for erythema, swelling, pruritus, rash, or other local reactions. Individuals with a clinically significant local reaction to either study preparation will be excluded before randomization, and the reason for exclusion will be documented.

**FIGURE 2:**
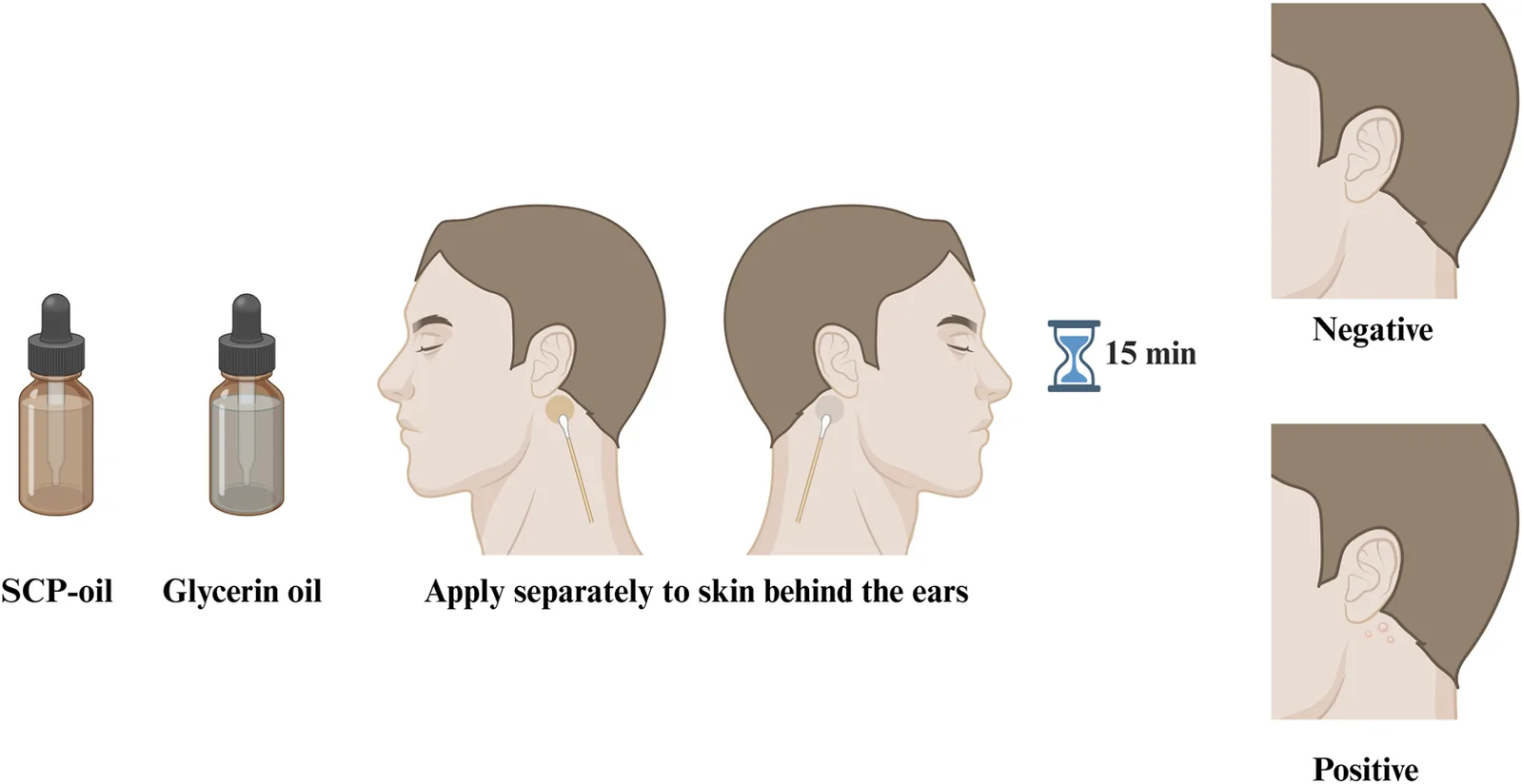
Procedure for pre-intervention allergy screening. Before randomization, SCP-oil and glycerin are applied separately to two postauricular skin sites and assessed after 15 min for signs of a local reaction. Participants with a positive reaction to either preparation will not proceed to randomization.

### 2.5 Randomization and blinding

Patients with ISSNHL who meet the eligibility criteria will be randomly assigned in a 1:1 ratio to the SCP-oil group or the glycerin placebo group using block randomization. An independent statistician will generate the randomization sequence using computer-generated random numbers and randomly varying block sizes, and allocation will be managed by an independent data manager or through a central randomization system. Allocation concealment will be ensured using sequentially numbered, opaque, sealed envelopes or an equivalent secure randomization procedure.

Throughout the trial, participants, outcome assessors (including audiologists responsible for audiometric evaluation), blinded data-management personnel, statisticians, and treating clinicians who are not involved in the preparation or administration of the study materials will remain blinded to treatment allocation. After a participant has been randomized, the allocation information will be disclosed only to the designated study nurse or pharmacist responsible for preparing the study materials. This individual will not participate in outcome assessment, data analysis, or efficacy evaluation. Neither participants nor other blinded investigators will be involved in the randomization process or have access to the allocation sequence. Participants will be informed that they may receive either of two study interventions. All study personnel will receive standardized training before trial initiation, and role separation will be maintained throughout the study.

To support blinding, the SCP-oil and glycerin control preparations will be provided in identical containers and matched as closely as possible with respect to volume, sponge size, appearance, and administration procedures. Nevertheless, glycerin differs from SCP-oil in viscosity, hydrophilicity, and odor, and complete sensory matching may not be achievable. Participants will not be shown or allowed to handle the study preparations during administration, and preparation of the study materials will be restricted to the designated unblinded study nurse or pharmacist. However, given the possibility of incomplete blinding, blinding will be formally evaluated using the Bang blinding index in the present trial. Participants will not be shown the study preparation during administration, and the potential effect of partial unblinding will be assessed formally at the end of the treatment period. At the end of the 2-week treatment period and before unblinding, participants and outcome assessors will be asked to indicate whether they believe the participant received SCP-oil, glycerin control, or whether they are uncertain. The Bang blinding index will be calculated separately for each randomized group and respondent type, together with corresponding 95% confidence intervals. The index will be interpreted as a continuous measure of departure from perfect blinding rather than as a binary indicator of blinding success or failure.

Emergency unblinding will be permitted only when knowledge of treatment allocation is essential for participant management, and each instance of emergency unblinding will be documented.

### 2.6 Sample size calculation

The assumptions used for the sample-size calculation were based on data from an unpublished preliminary observational cohort study conducted at Zhejiang Provincial Hospital of Chinese Medicine between May and June 2025. The cohort included 45 patients with ISSNHL, of whom 25 received conventional therapy plus SCP-oil ear-canal packing and 20 received conventional therapy alone. Using the prespecified definition of the primary outcome, the observed total markedly effective rates were 68% (17/25) in the SCP-oil group and 50% (10/20) in the comparison group.

The sample size was calculated for the comparison of two independent proportions using PASS version 2025. With a two-sided significance level of α = 0.05, 80% power, and a planned 1:1 allocation ratio, the calculation indicated that at least 116 participants were required per group. Allowing for a 10% loss to follow-up, the target sample size was set at 129 participants per group, for a total of 258 participants.

### 2.7 Interventions and clinical procedures

#### 2.7.1 Conventional therapy in both groups

Both groups will receive guideline-based standard therapy for ISSNHL during the 14-day treatment period. The standard regimen, developed with reference to relevant guidelines and published protocols, will include (Lee and Chung, 2024): systemic corticosteroid therapy with oral prednisone acetate, 50 mg once daily for 7 days, followed by tapering to discontinuation over the subsequent 7 days (Chandrasekhar et al., 2019); microcirculation therapy with oral betahistine mesylate, 12 mg three times daily; and (Kim et al., 2018) neurotrophic therapy with oral methylcobalamin, 0.5 mg three times daily (Editorial Board of Chinese Journal of Otorhinolaryngology Head and Neck Surgery, Society of Otorhinolaryngology Head and Neck Surgery, Chinese Medical Association, National Clinical Research Center for Otorhinolaryngologic Diseases, 2026).

#### 2.7.2 Preparation and quality control of SCP-oil

The SCP-oil used in this study will be prepared by the Department of Pharmacy, Zhejiang Provincial Hospital of Chinese Medicine, according to a predefined standard operating procedure. The crude *A. tatarinowii* rhizome will be purchased from Zhejiang Chinese Medical University Medical Pieces Co. Ltd. The batch number, geographic origin, certificate of analysis, and supplier qualifications will be documented. Before extraction, the crude rhizome will be authenticated by qualified pharmacy personnel, and the supplier’s certificate of analysis will be reviewed to confirm compliance with relevant quality standards.

SCP-oil will be obtained by supercritical CO2 extraction, with raw-material pretreatment, extraction parameters, extraction yield, and dispensing procedures standardized and documented. Quantitative GC–MS analysis of the designated SCP-oil batch intended for clinical use was performed by an independent third-party analytical laboratory using authentic reference standards. The measured contents of methyleugenol, α-asarone, and β-asarone were 0.13%, 0.73%, and 41.3%, respectively, corresponding to approximately 1.3, 7.3, and 413 mg/g of SCP-oil. For the present exploratory clinical trial, these quantitatively verified constituent levels were predefined as the provisional maximum acceptable amounts for the designated clinical batch. Accordingly, only SCP-oil batches meeting the following trial-specific acceptance criteria will be used for clinical administration: methyleugenol ≤ 0.13%, α-asarone ≤ 0.73%, and β-asarone ≤ 41.3%. Methyl isoeugenol was additionally quantified at 0.14% as part of the chemical characterization of the clinical batch.

Based on the planned administration volume of approximately 0.04 mL/day and an assumed essential-oil density of approximately 1 g/mL, the corresponding nominal administered amounts were estimated to be approximately 0.052 mg/day for methyleugenol, 0.292 mg/day for α-asarone, and 16.52 mg/day for β-asarone. Over the 14-day treatment period, corresponding to a scheduled cumulative administration volume of approximately 0.56 mL, the cumulative nominal amounts were estimated to be approximately 0.73, 4.09, and 231.28 mg, respectively. These values represent the quantities applied locally to the external auditory canal rather than confirmed systemic exposure because route-specific systemic absorption will not be directly measured in the present trial.

As the present study is a single-center exploratory trial, all participants will receive SCP-oil from the same designated production batch that has undergone predefined quality-control assessment and quantitative characterization. Therefore, the current quality-control strategy focuses on ensuring consistency and controlling constituent exposure within this clinical trial rather than establishing universal manufacturing specifications applicable across multiple production batches. If further clinical development is supported, broader constituent-content specifications and batch-release criteria will be established and validated using multiple independent production batches. If the findings of the present study support further clinical development, formal constituent-content specifications and batch-release criteria will be established and validated using data from multiple independent production batches before initiation of a subsequent multicenter trial.

The same qualified production batch of SCP-oil will be used for all participants throughout the trial to minimize inter-participant variability attributable to production-batch differences. The oil will be stored in sealed amber containers at low temperature and protected from light.

#### 2.7.3 SCP-oil ear-canal packing

In the SCP-oil group, a gelatin sponge measuring 2 × 2 mm will be impregnated with approximately 0.04 mL of SCP-oil for each daily application, resulting in a total cumulative volume of approximately 0.6 mL over the 14-day treatment period. The oil-soaked sponge will be placed at the junction of the cartilaginous and bony portions of the external auditory canal in the affected ear and replaced once daily. Ear-canal packing will be continued for 14 consecutive days, in accordance with the predefined treatment period All procedures will be performed by a licensed physician. The ear-canal packing procedure is illustrated in Figure 3.

**FIGURE 3:**
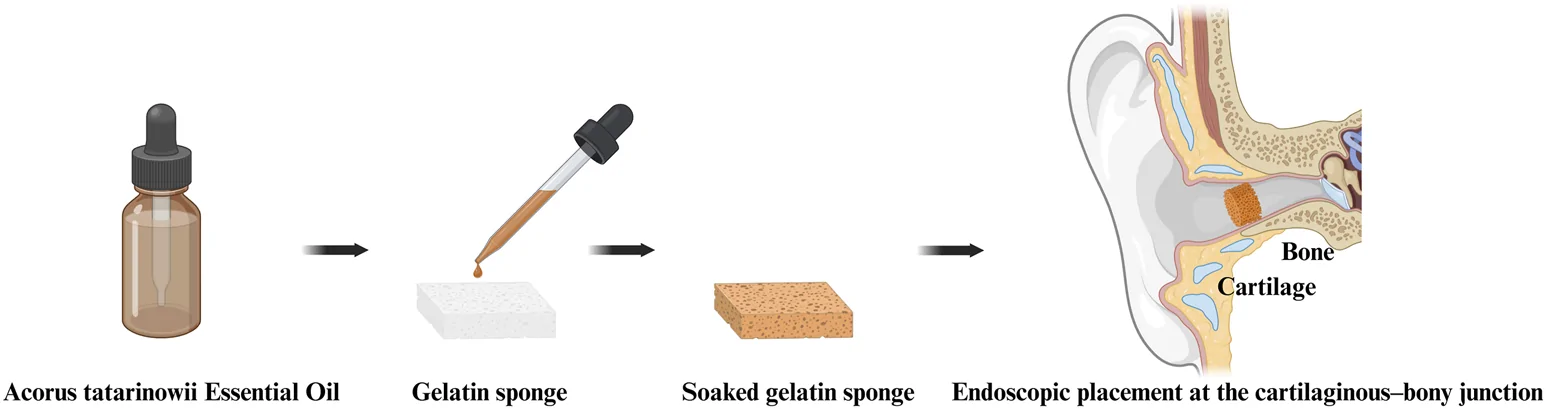
Flowchart showing the placement of a gelatine sponge soaked in SCP-oil in the external auditory canal. A 2 × 2 mm gelatin sponge is impregnated with approximately 0.04 mL of SCP-oil and placed endoscopically at the junction of the cartilaginous and bony portions of the affected external auditory canal. SCP-oil, Acorus tatarinowii Schott essential oil.

The cumulative dose of approximately 0.6 mL was determined using an empirical approach informed by the traditional external application to the ear of *A. tatarinowii* in traditional Chinese medicine. In the traditional ear-packing regimen on which the present intervention was based, approximately 30 g of *A. tatarinowii* rhizome is used over a treatment course, and its volatile-oil content has been reported to range from 2% to 3%. Using the conservative estimate of 2%, 30 g of crude rhizome would contain approximately 0.6 g of essential oil. Assuming an essential-oil density of approximately 1 g/mL, this amount corresponds to approximately 0.6 mL and served as a pragmatic reference for selecting the cumulative dose for the 14-day treatment period in the present study. The selected regimen was also informed by its feasibility and short-term tolerability based on our preliminary clinical experience and ongoing prospective observational cohort. Because no formal dose-ranging study has been conducted, this dose should be regarded as an empirical dose for the present exploratory trial rather than an established optimal dose.

The 14-day treatment duration was selected to coincide with the protocol-defined course of conventional therapy for ISSNHL and primary outcome assessment at week 2, allowing SCP-oil ear-canal packing to be integrated with standard treatment without extending the intervention period.

#### 2.7.4 Placebo ear-canal packing

In the control group, pharmaceutical-grade glycerin will be used as the placebo to impregnate a gelatin sponge of identical size (2 × 2 mm). Pharmaceutical-grade glycerin was selected as the placebo vehicle because it has relatively limited intrinsic pharmacological activity, a long history of use in otologic preparations, and a well-established safety profile for application within the external auditory canal. It can adequately impregnate the gelatin sponge and support a standardized ear-canal packing procedure while minimizing the likelihood of producing antimicrobial, anti-inflammatory, antioxidant, or other nonspecific local therapeutic effects. Therefore, glycerin is expected to have a low probability of independently influencing hearing recovery or other clinical outcomes, making it an appropriate placebo vehicle for more accurately estimating the specific efficacy and safety of SCP-oil.

The glycerin placebo will be matched to SCP-oil as closely as possible with respect to administered volume, sponge size, packaging, appearance, administration site, replacement schedule, and administration procedures. The cumulative volume administered in each group will be approximately 0.6 mL over the 14-day treatment period. The glycerin-soaked sponge will be placed at the junction of the cartilaginous and bony portions of the external auditory canal in the affected ear and replaced once daily for 14 consecutive days. All procedures will be performed by a licensed physician.

#### 2.7.5 Concomitant care and rescue treatment

Rescue intratympanic corticosteroid treatment will be available to participants in either randomized group who have inadequate hearing recovery during the intervention period. Eligibility for rescue treatment will be assessed by a qualified otologist using prespecified clinical criteria applied equally to both groups, including insufficient hearing-threshold improvement or persistent severe or profound hearing loss. The decision to initiate rescue treatment will be based solely on clinical need and, wherever feasible, will be made by an otologist who remains blinded to treatment allocation.

Rescue treatment will be administered in accordance with institutional standard operating procedures and current clinical guidelines. The corticosteroid administered, its concentration, administration volume, injection frequency, total number of injections, dates of administration, indication for rescue treatment, and any associated adverse events will be documented. Participants who receive rescue treatment will remain in the trial and continue to complete all scheduled outcome assessments. The assigned ear-canal intervention will also be continued.

#### 2.7.6 Criteria for discontinuation of the study intervention and trial stopping rules

The assigned ear-canal intervention may be discontinued early if pure-tone audiometry confirms that hearing thresholds at the affected frequencies have returned to the premorbid level; if the participant requests discontinuation; if clinically significant intolerance, an adverse event, or another medical condition renders continuation of the intervention inappropriate; or if discontinuation is considered necessary by the investigator to be in the participant’s best clinical interest. If early hearing recovery is suspected during the intervention period, an unscheduled pure-tone audiometry assessment will be performed to determine whether the discontinuation criterion has been met. Discontinuation of the assigned intervention will not constitute withdrawal from the trial. Participants will remain in their randomized groups and will be encouraged to complete all scheduled efficacy and safety assessments unless they withdraw consent.

At the trial level, recruitment and administration of the study interventions will be temporarily suspended if any of the following occurs (Lee and Chung, 2024): an unexpected serious adverse event that is considered probably or definitely related to either study preparation (Chandrasekhar et al., 2019); two or more participants experience the same severe treatment-related adverse event (Kim et al., 2018); two or more participants develop suspected treatment-related ototoxicity; or (Stachler et al., 2012) a study-preparation batch fails any protocol-defined quality-control requirement relating to identity, chemical characterization, stability, contamination, or other pharmaceutical-quality requirements.

When a trial-level stopping criterion is met, further recruitment and administration of the study interventions will be suspended immediately. The available safety data will be reviewed by the principal investigator together with an independent safety reviewer or safety-monitoring committee within 72 h. The institutional ethics committee will be informed in accordance with applicable requirements. The trial may be resumed without modification, resumed after protocol modification, or permanently terminated on the basis of the safety review and ethics approval.

### 2.8 Outcome measurement

All outcome assessments will be performed at baseline and at week 2 by trained physicians or audiologists using standardized procedures. An additional safety follow-up will be conducted at week 4. All study data will be recorded using standardized procedures. The primary audiological outcome will be the total markedly effective rate at week 2. Secondary audiological outcomes will include the mean improvement in hearing thresholds across the affected frequencies and the distribution of the four hearing-recovery categories. The affected frequencies will be identified among the tested frequencies of 0.125, 0.25, 0.5, 1, 2, 4, and 8 kHz. Patient-reported secondary outcomes will include the VAS for tinnitus severity, the SDS, and the PSQI.

#### 2.8.1 Primary outcome

Hearing recovery will be evaluated in accordance with the *Guideline for Diagnosis and Treatment of Sudden Sensorineural Hearing Loss (2026 Practical Edition).* The primary outcome will be the total markedly effective rate of hearing recovery at week 2. Hearing recovery will be classified as follows (Lee and Chung, 2024): cured, hearing thresholds at the affected frequencies return to normal, reach the level of the contralateral healthy ear, or return to the premorbid level (Chandrasekhar et al., 2019); markedly effective, the mean hearing threshold at the affected frequencies improves by > 30 dB (Kim et al., 2018); effective, the mean hearing threshold at the affected frequencies improves by 15–30 dB, inclusive; and (Stachler et al., 2012) ineffective, the mean hearing threshold at the affected frequencies improves by < 15 dB. The total markedly effective rate will be calculated as follows: total markedly effective rate of hearing recovery = (number of cured cases + number of markedly effective cases)/number of participants with evaluable week-2 hearing-recovery data × 100%.

#### 2.8.2 Secondary outcomes

Secondary outcomes will be assessed at baseline and week 2 (Lee and Chung, 2024). The mean hearing-threshold improvement: The mean hearing-threshold improvement will be calculated as the mean absolute change from baseline to week 2 across the affected frequencies, selected from the tested frequencies of 0.125, 0.25, 0.5, 1, 2, 4, and 8 kHz. A positive value will indicate an improvement in hearing threshold (Chandrasekhar et al., 2019). Hearing-recovery categories: At week 2, the numbers and proportions of participants classified as cured, markedly effective, effective, or ineffective will be reported separately for each treatment group (Kim et al., 2018). Tinnitus VAS: Participants will rate tinnitus severity on a 0–10 scale (0 indicates no tinnitus and 10 indicates the worst imaginable or intolerable tinnitus), higher scores indicating more severe tinnitus (Stachler et al., 2012). SDS: Depressive symptoms will be evaluated using the 20-item SDS, which uses a four-point response format; Raw scores will be converted to standard scores, with scores <53 considered normal, 53–62 indicating mild depression, 63–72 indicating moderate depression, and >72 indicating severe depression (Rodríguez-Izquierdo et al., 2025). PSQI: Sleep quality will be assessed using the PSQI with a global score ranging from 0 to 21, higher scores indicating poorer sleep quality.

### 2.9 Safety evaluation and adverse events (AEs)

Available toxicological evidence has raised safety concerns regarding herbal medicinal products containing asarone and methyleugenol, particularly following repeated, prolonged, or systemic exposure (Liu et al., 2024; Uebel et al., 2021; Chen et al., 2024). The relevance of these findings to short-term local administration via the external auditory canal remains uncertain, and systemic absorption cannot be excluded. Available toxicological reference values for α-asarone, β-asarone, and methyleugenol are derived predominantly from animal oral or systemic exposure studies and are therefore regarded as contextual safety references rather than direct safety limits for external auditory-canal administration.

In the present study, approximately 0.04 mL of SCP-oil is retained locally on a 2 × 2 mm gelatin sponge within the external auditory canal. Gelatin-based matrices have been investigated as localized drug-delivery vehicles capable of retaining incorporated substances and modulating their release (Young et al., 2005). Accordingly, the gelatin sponge serves as a localized carrier and may limit rapid dispersion of the formulation from the administration site. However, the extent of systemic absorption of α-asarone, β-asarone, and methyleugenol through this administration route remains unknown. Therefore, potential systemic exposure and toxicological risk will be considered in the safety evaluation.

All AEs will be monitored prospectively in both groups from the first administration of the assigned study preparation through the 2-week intervention period and until the safety follow-up at week 4. AEs will be identified through inpatient observation, participant self-report, structured physician assessment, examination at each sponge replacement, and scheduled follow-up. AEs will be graded as mild, moderate, or severe based on symptom intensity, the need for medical intervention, interference with daily activities, and the impact on continuation of the study intervention. Serious adverse events (SAEs) will also be recorded and managed in accordance with standard clinical practice and applicable regulatory requirements.

Local otologic AEs of particular interest will include external auditory canal irritation or minor trauma, burning sensation, pruritus, erythema, edema, rash, otalgia, aural fullness, otorrhea, local abrasion or bleeding, allergic contact dermatitis, secondary otitis externa, clinically significant or prolonged canal occlusion, and discomfort associated with daily sponge replacement. Potential formulation-related intolerance or hypersensitivity reactions will also be recorded. In addition, participants will be monitored for newly developed or worsening auditory, vestibular, or systemic symptoms, including worsening tinnitus, dizziness, vertigo, and imbalance.

At each daily sponge replacement, trained study personnel will remove the sponge and examine the external auditory canal and tympanic membrane for evidence of irritation, trauma, dermatitis, infection, abnormal discharge, canal occlusion, or other clinically significant abnormalities. Participants will also be systematically asked whether they have experienced local discomfort, otalgia, burning sensation, pruritus, aural fullness, and any newly developed or worsening auditory, vestibular, or systemic symptoms. Because both groups will undergo the same ear-canal packing and daily sponge-replacement procedures, procedure-related AEs will be assessed identically in both groups.

Sterile, single-use materials and standardized aseptic procedures will be used throughout the intervention period, and the gelatin sponge will be carefully inserted and removed to minimize local trauma. If clinically significant intolerance, pain, dermatitis, infection, persistent canal occlusion, or other AEs occur, ear-canal packing will be temporarily suspended or permanently discontinued, as clinically appropriate. Participants will undergo otologic evaluation and receive appropriate medical management.

For each AE, the onset date, duration, severity, management, outcome, and relationship to the assigned study intervention will be documented. Follow-up for the event will continue until resolution or stabilization. Trial enrollment and ongoing interventions will be reviewed if unexpected serious safety signals emerge.

### 2.10 Data management and quality control

A standardized case report form (CRF) will be established in a secure electronic data capture system before study initiation. Trained investigators will enter data into the CRF from the original source documents according to predefined coding rules. Each participant will be assigned a unique study identification number, and personal identifiers will be stored separately from the research database to protect participant confidentiality. Data quality will be ensured through built-in range checks, logic checks, and double data entry by two independent trained data-entry personnel. Any discrepancies will be resolved by comparison with the original source documents, and all corrections will be documented to maintain a complete audit trail. Outcome assessments will be performed by assessors who are blinded to treatment allocation. Before trial initiation, all study personnel will receive standardized training to ensure consistency in data collection, intervention delivery, outcome assessment, and adverse-event reporting. Study documents and treatment records will be stored securely with restricted access. In addition, regular quality-control reviews and investigator meetings will be conducted every 3 months to monitor protocol adherence, data completeness, follow-up status, and safety reporting. Participants will receive telephone reminders before scheduled visits to improve follow-up attendance. Reasons for trial withdrawal or loss to follow-up will be recorded in detail.

### 2.11 Statistical analysis

Statistical analyzes will be performed using Stata software. Participants will be analyzed according to their randomized treatment allocation. No participant will be excluded because of treatment nonadherence or discontinuation; however, each outcome-specific analysis will include only participants with sufficient data to derive the relevant outcome. A per-protocol analysis will be conducted as a sensitivity analysis. Continuous variables will be summarized as the mean and standard deviation or the median and interquartile range, as appropriate, whereas categorical variables will be presented as frequencies and percentages. Distributional assumptions will be assessed using graphical methods and the Shapiro–Wilk test. Baseline characteristics will be summarized by treatment group; formal significance testing of baseline differences will not be used to determine treatment efficacy.

The primary outcome is the total markedly effective rate at week 2, defined as the proportion of participants classified as cured or markedly effective. The unadjusted between-group comparison will be performed using the chi-square test or Fisher’s exact test, as appropriate. The primary treatment effect will be estimated as a risk ratio with a 95% confidence interval using modified Poisson regression with robust variance estimation. The distribution of the four hearing-recovery categories will be summarized by treatment group and compared using the chi-square test or Fisher’s exact test, as appropriate. Continuous secondary outcomes, including mean hearing-threshold improvement, tinnitus VAS, SDS, and PSQI, will be analyzed using analysis of covariance. Adjusted between-group differences and 95% confidence intervals will be reported.

The extent, patterns, and reasons for missing data will be summarized by treatment group. Audiological outcomes will not be imputed; participants without sufficient paired baseline and week-2 audiometric data will be excluded only from the relevant analysis. Missing week-2 values for non-audiological secondary outcomes will be handled using multiple imputation by chained equations under the missing-at-random assumption, with estimates combined using Rubin’s rules and complete-case analyzes performed as sensitivity analyzes.

Intratympanic corticosteroid treatment initiated before the week-2 assessment will be considered an intercurrent event. The primary analysis will adopt a treatment-policy strategy, whereby participants will remain in their randomized groups and all available week-2 outcomes will be analyzed regardless of rescue-treatment use. In a prespecified sensitivity analysis of the primary outcome, participants who receive intratympanic corticosteroids before week 2 will be classified as nonresponders. Exploratory subgroup analyzes according to baseline hearing-loss subtype will be performed using treatment-by-subtype interaction terms. All statistical tests will be two-sided, and a P value <0.05 will be considered statistically significant.

## 3 Discussion

Current therapeutic options for ISSNHL remain suboptimal. Although corticosteroids remain the cornerstone of treatment, clinical outcomes are highly variable, and a substantial proportion of patients fail to achieve satisfactory hearing recovery despite timely intervention (Chandrasekhar et al., 2019). Interpretation of treatment effects is further complicated by the uncertain efficacy of several adjunctive and nonsteroidal therapies (Rodríguez-Izquierdo et al., 2025; Chaushu et al., 2023). In addition, some adjunctive approaches are invasive or difficult to implement in routine practice (Lee and Chung, 2024; Mirian and Ovesen, 2020; Singh and Kumar Irugu, 2020). Against this background, SCP-oil administered via ear-canal packing may offer several practical advantages, including a noninvasive mode of delivery, ease of administration, and the potential for improved patient adherence. It may also provide a feasible framework for the standardization and modern clinical evaluation of external therapies used in traditional Chinese medicine Accordingly, this randomized, double-blind, placebo-controlled trial is warranted to generate more rigorous evidence regarding its efficacy and safety.

Another important strength of this protocol is its emphasis on standardization and reproducibility. External therapies in traditional Chinese medicine may be limited by heterogeneity in formulation, administration, and quality control, which may undermine interpretability and reproducibility across studies. To address these concerns, the present study prespecifies the source of SCP-oil, preparation procedures, qualitative GC-MS characterization, batch-consistency measures, dosing regimen, administration frequency, treatment duration, and procedures for adverse-event monitoring (Liu et al., 2024; Uebel et al., 2021; Bechis et al., 2023; Capetti et al., 2021). By clearly defining these methodological elements in advance, the study seeks not only to improve internal consistency, but also to provide a reproducible operational framework for future clinical evaluations of similar external interventions.

Regarding audiological efficacy, the total markedly effective rate was selected as the primary outcome because it offers a clinically interpretable measure of the proportion of participants achieving complete hearing recovery or marked hearing improvement. This composite endpoint also accounts for differences in baseline hearing-loss severity: participants with relatively mild hearing loss may achieve complete recovery despite a modest absolute improvement in hearing thresholds, whereas those with severe hearing loss may experience an improvement of more than 30 dB without returning to normal hearing. To complement this categorical primary outcome, the mean improvement in hearing thresholds across the affected frequencies has been included as a continuous secondary outcome, and the distribution across the four hearing-recovery categories will also be reported. Nevertheless, categorical assessment depends on prespecified thresholds and may result in some loss of information. The total markedly effective rate should therefore be interpreted together with the continuous hearing outcome and the individual hearing-recovery categories. In addition to objective audiometric measures, this study evaluates tinnitus severity, sleep quality, and depressive symptoms. Such a broader assessment is clinically meaningful, as ISSNHL is frequently accompanied by tinnitus-related distress, depressive symptoms, and sleep disturbance (Chen et al., 2013). Previous cohort studies have also reported associations between ISSNHL and affective disorders, such as depression and anxiety (Kim et al., 2018), while nationwide cohort data suggest that ISSNHL is associated with an increased risk of insomnia, particularly in patients with comorbid tinnitus, depression, or anxiety (Yeo et al., 2022). Incorporating these outcomes may therefore provide a more comprehensive assessment of patient-centered and clinically meaningful outcomes.

Beyond these clinical and methodological features, the biological rationale for the intervention also merits consideration. From the perspective of traditional Chinese medicine, *A. tatarinowii* has historically been used to “open the orifices” and has been documented in the treatment of disorders involving hearing and consciousness, providing a historical rationale for exploring its external application in ear disorders (Castañeda et al., 2019; Wang et al., 2023). From a modern pharmacological perspective, preclinical studies suggest that SCP-oil and its major constituents may exert anti-inflammatory, antioxidant, and neuroprotective effects by modulating inflammatory and oxidative stress-related pathways (Balakrishnan et al., 2022; Xu et al., 2023; Lim et al., 2014). Complementing these findings, preliminary animal experiments conducted by our group have provided qualitative evidence of possible cochlear exposure following external auditory canal administration, supporting the biological plausibility of this delivery route. Taken together, the traditional context, existing pharmacological evidence, and preliminary animal observations provide a rationale for evaluating SCP-oil ear-canal packing as a potential noninvasive adjunct to conventional therapy for ISSNHL. Nevertheless, the available evidence remains preliminary and does not establish the mechanism of action or clinical efficacy of SCP-oil in ISSNHL. In particular, the precise transport pathway across the intact tympanic membrane, pharmacokinetic profile, cochlear drug concentrations, dose–exposure relationship, and the ability to achieve therapeutically meaningful cochlear concentrations remain to be determined. Further pharmacokinetic and mechanistic studies are therefore warranted to clarify inner-ear delivery, biological effects, and potential therapeutic relevance of externally administered SCP-oil.

Several limitations should be considered. First, as a single-center trial, this study will recruit participants from only one institution. Differences in patient characteristics, referral patterns, clinical practices and healthcare settings across institutions may therefore limit the generalizability of the findings to other populations and settings. If the findings are favorable, confirmation in an adequately powered multicenter randomized controlled trial will be required before broader clinical application (Austin, 2011; Chesnaye et al., 2022). Second, spontaneous hearing recovery may occur in ISSNHL, particularly early after symptom onset, and may contribute to the hearing gains observed in both groups. Although randomisation and placebo control are expected to balance the effects of spontaneous recovery and related prognostic factors between the groups, they cannot completely eliminate the possibility of chance imbalances. Any estimated treatment effect should therefore be interpreted primarily on the basis of between-group differences rather than within-group improvement alone. Third, because SCP-oil ear-canal packing will be administered as an adjunct to protocol-defined conventional treatment, the observed outcomes may also be influenced by baseline disease severity, the interval between symptom onset and treatment initiation, and variations in treatment adherence. These baseline and treatment-related factors will therefore be recorded systematically and, where appropriate, accounted for in the statistical analyses. Besides, the 4-week follow-up period is insufficient to determine the long-term durability of treatment effects or the risk of recurrence. Finally, glycerin was selected as a pharmacologically more inert placebo to reduce the risk that the control preparation itself would exert antimicrobial, anti-inflammatory, antioxidant, or other nonspecific local effects. However, glycerin is hydrophilic and more viscous than SCP-oil and differs in odor, which may affect sponge saturation, release characteristics, and sensory matching. These differences could contribute to differential local exposure or partial unblinding. Standardized preparation and administration procedures and formal assessment with the Bang blinding index are intended to mitigate and quantify these risks, but they cannot eliminate them. This possibility should therefore be considered when interpreting the efficacy and safety findings of the trial.

Overall, this randomized, double-blind, placebo-controlled trial is expected to contribute in two complementary ways. Clinically, it will provide more rigorous evidence on the efficacy and safety of SCP-oil ear-canal packing as an adjunct to conventional therapy for ISSNHL. Methodologically, it may provide a standardized and reproducible framework for evaluating traditional external interventions in contemporary clinical research. If the findings are favorable, a larger, adequately powered, multicenter randomized controlled trial will be needed to confirm the efficacy and safety of the intervention across diverse patient populations and clinical settings, thereby strengthening the generalizability and robustness of the evidence. Complementary pharmacokinetic, otologic drug-delivery, and biomarker studies will also be required to clarify the underlying mechanisms and support the further clinical development of this intervention.
