# Supplementary material for: Adjunctive Acorus tatarinowii essential-oil ear-canal packing for idiopathic sudden sensorineural hearing loss: study protocol for a single-center, randomized, double-blind, placebo-controlled clinical trial
Source: Front Pharmacol. 2026 Sep 30;17:1875920. doi: 10.3389/fphar.2026.1875920 (PMC13662148; doi:10.3389/fphar.2026.1875920)
Supplement: Supplementary file 2 [file Table2.doc]

**SPIRIT-TCM Extension 2018 Checklist: Recommended items to address in a clinical trial protocol and related documents***

| Section/item | Item No | Description | **Section Heading** |
| --- | --- | --- | --- |
| **Administrative information** | | |  |
| Title | 1a | Specify the patient population in terms of 1) a WM-defined disease, 2) a WM-defined disease with a specific TCM Pattern, or 3) a TCM Pattern. | Introduction |
|  | 1b | Specify the intervention, in terms of 1) CHM formula, 2) acupuncture, 3) moxibustion, or 4) other TCM therapy(ies). | Interventions and clinical procedures |
| Trial registration | 2a | Trial identifier and registry name. If not yet registered, name of intended registry | Study design |
|  | 2b | All items from the World Health Organization Trial Registration Data Set | Study design |
| Protocol version | 3 | Date and version identifier | The 1th version dated on 15th March 2026. |
| Funding | 4 | Sources and types of financial, material, and other support | Funding |
| Roles and responsibilities | 5a | Names, affiliations, and roles of protocol contributors | Title page, Author contributions |
|  | 5b | Name and contact information for the trial sponsor | N/A  Not applicable in this trial |
|  | 5c | Role of study sponsor and funders, if any, in study design; collection, management, analysis, and interpretation of data; writing of the report; and the decision to submit the report for publication, including whether they will have ultimate authority over any of these activities | N/A  Not applicable in this trial |
|  | 5d | Composition, roles, and responsibilities of the coordinating centre, steering committee, endpoint adjudication committee, data management team, and other individuals or groups overseeing the trial, if applicable (see Item 21a for data monitoring committee) | N/A |
| Introduction |  |  |  |
| Background and rationale | 6a. 1 | Provide the background and rationale of the research question with TCM theory. | Introduction |
|  | 6a. 2 | Describe the rationale of the utilized TCM interventions with references. | Introduction |
|  | 6a. 3 | Provide the rationale of adding experimental TCM interventions if WM intervention is used as basic or combined remedy. If possible, the potential interaction between WM intervention and TCM intervention (especially for CHM) should also be explained with related reference(s). | Introduction |
|  | 6b | Describe the rationale and principle(s) for selecting comparators corresponding to certain interventions (i.e. CHM formula, acupuncture, moxibustion or other TCM interventions), considering 1) comparable with tested intervention; 2) success of blinding. | Interventions and clinical procedures |
| Objectives | 7 | State the objectives or hypotheses regarding the specific TCM intervention for 1) a WM-defined disease, 2) a WM-defined disease with a specific TCM Pattern or 3) a TCM Pattern. | Inclusion criteria |
| Trial design | 8 | Description of trial design including type of trial (eg, parallel group, crossover, factorial, single group), allocation ratio, and framework (eg, superiority, equivalence, noninferiority, exploratory) | Study design |
| Methods: Participants, interventions, and outcomes | | |  |
| Study setting | 9 | Description of study settings (eg, community clinic, academic hospital) and list of countries where data will be collected. Reference to where list of study sites can be obtained | Study design |
| Eligibility criteria | 10a | State whether participants with a specific TCM Pattern will be recruited, in terms of 1) diagnostic criteria, and 2) inclusion and exclusion criteria. All criteria utilized should be universally recognized, or reference(s) where detailed explanation can be found should be given. | Not applicable in this trial |
|  | 10b | Descriptions of the roles, qualifications and other relevant experience of the researchers (e.g. participant screeners, care providers, outcome assessors, data analysts) in TCM research are recommended. | Randomization and blinding, SCP-oil ear-canal packing |
|  | 10c | Descriptions of the qualification and relevant experience of study center(s) involved in a TCM trial are recommended. | N/A |
| Interventions | 11a. 1 | Interventions for the experimental group(s) with sufficient detail to allow replication. | Preparation and quality control of SCP-oil, SCP-oil ear-canal packing |
|  | 11a.1A | CHM formulae  For fixed CHM formulae  1. Name, source and dosage form (e.g. decoction, granules, powder, pills).  2. Name, source, processing method and dosage of each medical substance. Name of all substances should be presented in at least two types of languages: Chinese (Pinyin), Latin or English. Names of the parts of the substances used should be specified.  3. Authentication method of each ingredient, and how, when, where, by whom it will be conducted.  4. Production method of the formula.  5. Quality control of each ingredient and the whole formula.  6. Safety assessment of the formula, containing heavy metals and toxic elements test, pesticide residue test, microbial limit test, acute/chronic toxicity test.  7. Dosage of the formula, and how the dosage was determined.  8. Administration route (e.g. oral, external).  For individualized CHM formulae  1. As for fixed CHM formulae, refer to fixed formulae Point 1-8 listed above.  2. Additional information: how, when and by whom the formula will be modified. | Preparation and quality control of SCP-oil |
|  | 11a.1B | Acupuncture | N/A  Not applicable in this trial |
|  | 11a.1C | Moxibustion | N/A  Not applicable in this trial |
|  | 11a.2 | Interventions for the control group(s) with sufficient detail to allow replication. | Preparation and quality control of SCP-oil, SCP-oil ear-canal packing |
|  | 11a.2A | CHM formulae Placebo control  1. Name and dosage of each ingredient.  2. Description of the similarity of placebo with intervention (e.g. color, smell, taste, appearance, packing).  3. Quality control and safety surveillance, if any.  4. Administration route, dosage and regimen.  5. Production information: when, where, how and by whom the placebo was produced. | Placebo ear-canal packing |
|  | 11a.2B | Acupuncture  Sham acupuncture or acupuncture-like control | N/A  Not applicable in this trial |
|  | 11a.2C | Moxibustion  Sham moxibustion or moxibustion-like control | N/A  Not applicable in this trial |
|  | 11b | Criteria for discontinuing or modifying allocated interventions for a given trial participant (eg, drug dose change in response to harms, participant request, or improving/worsening disease) | Safety evaluation and adverse events(AEs) |
|  | 11c | Strategies to improve adherence to intervention protocols, and any procedures for monitoring adherence (eg, drug tablet return, laboratory tests) | N/A |
|  | 11d | Descriptions of other interventions that will be administrated to experimental and/or control groups are recommended (e.g. rescue interventions), with enough details to allow replication. | Safety evaluation and adverse events(AEs) |
| Outcomes | 12a | Provide the rationale of TCM-related indexes as outcomes (e.g. the change of degree and scope of symptoms and signs related to Pattern differentiation). | N/A  Not applicable in this trial |
|  | 12b | Provide the details of the TCM-related outcomes assessment, including i) the measuring methods and standard (e.g. frequency, severity rating scale of symptoms and signs, verified Pattern questionnaire, time points for assessment and corresponding rationale), ii) assessor qualification (e.g. relevant assessment experience, years in clinical practice), iii) methods used to enhance the quality of assessment (e.g.multiple repeated observation, training of assessors), and iv) related reference(s). | N/A  Not applicable in this trial |
| Participant timeline | 13 | Time schedule of enrolment, interventions (including any run-ins and washouts), assessments, and visits for participants. A schematic diagram is highly recommended (see Figure) | Study design, Figure 1, Table 1 |
| Sample size | 14 | Estimated number of participants needed to achieve study objectives and how it was determined, including clinical and statistical assumptions supporting any sample size calculations | Sample size calculation |
| Recruitment | 15 | Strategies for achieving adequate participant enrolment to reach target sample size | Sample size calculation |
| **Methods: Assignment of interventions (for controlled trials)** | | |  |
| Allocation: |  |  |  |
| Sequence generation | 16a | Method of generating the allocation sequence (eg, computer-generated random numbers), and list of any factors for stratification. To reduce predictability of a random sequence, details of any planned restriction (eg, blocking) should be provided in a separate document that is unavailable to those who enrol participants or assign interventions | Randomization and blinding |
| Allocation concealment mechanism | 16b | Mechanism of implementing the allocation sequence (eg, central telephone; sequentially numbered, opaque, sealed envelopes), describing any steps to conceal the sequence until interventions are assigned | Randomization and blinding |
| Implementation | 16c | Who will generate the allocation sequence, who will enrol participants, and who will assign participants to interventions | Study design, Randomization and blinding |
| Blinding (masking) | 17a | Who will be blinded after assignment to interventions (eg, trial participants, care providers, outcome assessors, data analysts), and how | Randomization and blinding |
|  | 17b | If blinded, circumstances under which unblinding is permissible, and procedure for revealing a participant’s allocated intervention during the trial | Randomization and blinding |
| **Methods: Data collection, management, and analysis** | | |  |
| Data collection methods | 18a | When trial targeting on TCM Pattern, or a WM-defined disease with a specific TCM  Pattern, baseline data about TCM Pattern should be provided. | N/A  Not applicable in this trial |
|  | 18b | Plans to promote participant retention and complete follow-up, including list of any outcome data to be collected for participants who discontinue or deviate from intervention protocols | Data management and quality control |
| Data management | 19 | Plans for data entry, coding, security, and storage, including any related processes to promote data quality (eg, double data entry; range checks for data values). Reference to where details of data management procedures can be found, if not in the protocol | Data management and quality control |
| Statistical methods | 20a | Statistical methods for analysing primary and secondary outcomes. Reference to where other details of the statistical analysis plan can be found, if not in the protocol | Statistical analysis |
|  | 20b | Methods for any additional analyses (eg, subgroup and adjusted analyses) | Statistical analysis |
|  | 20c | Definition of analysis population relating to protocol non-adherence (eg, as randomised analysis), and any statistical methods to handle missing data (eg, multiple imputation) | Statistical analysis |
| **Methods: Monitoring** | | |  |
| Data monitoring | 21a | Composition of data monitoring committee (DMC); summary of its role and reporting structure; statement of whether it is independent from the sponsor and competing interests; and reference to where further details about its charter can be found, if not in the protocol. Alternatively, an explanation of why a DMC is not needed. | N/A  In this trial, Ethics Committees of related institutes and Department of Health play a role to monitor the data. |
|  | 21b | Description of any interim analyses and stopping guidelines, including who will have access to these interim results and make the final decision to terminate the trial | N/A |
| Harms | 22 | Plans for collecting, assessing, reporting, and managing solicited and spontaneously reported adverse events and other unintended effects of trial interventions or trial conduct | Safety evaluation and adverse events(AEs) |
| Auditing | 23 | Frequency and procedures for auditing trial conduct, if any, and whether the process will be independent from investigators and the sponsor | N/A |
| Ethics and dissemination | | |  |
| Research ethics approval | 24 | Plans for seeking research ethics committee/institutional review board (REC/IRB) approval | Ethics statement |
| Protocol amendments | 25 | Plans for communicating important protocol modifications (eg, changes to eligibility criteria, outcomes, analyses) to relevant parties (eg, investigators, REC/IRBs, trial participants, trial registries, journals, regulators) | N/A |
| Consent or assent | 26a | Who will obtain informed consent or assent from potential trial participants or authorised surrogates, and how (see Item 32) | Recruitment process |
|  | 26b | Additional consent provisions for collection and use of participant data and biological specimens in ancillary studies, if applicable | N/A  Not applicable in this trial |
| Confidentiality | 27 | How personal information about potential and enrolled participants will be collected, shared, and maintained in order to protect confidentiality before, during, and after the trial | Data management and quality control |
| Declaration of interests | 28 | Financial and other competing interests for principal investigators for the overall trial and each study site | Conflict of interest |
| Access to data | 29 | Statement of who will have access to the final trial dataset, and disclosure of contractual agreements that limit such access for investigators | Data management and quality control |
| Ancillary and post-trial care | 30 | Provisions, if any, for ancillary and post-trial care, and for compensation to those who suffer harm from trial participation | Safety evaluation and adverse events(AEs) |
| Dissemination policy | 31a | Plan for raw data sharing, if any. The contents should contain: i) when the data will become available; ii) how the data will be shared iii) what data in particular will be shared; iv) who could acquire the data; v) through what access data will be shared. | N/A |
|  | 31b | Authorship eligibility guidelines and any intended use of professional writers | N/A  Not applicable in this trial |
|  | 31c | Plans, if any, for granting public access to the full protocol, participant-level dataset, and statistical code | N/A  Not applicable in this trial |
| Appendices |  |  |  |
| Informed consent materials | 32 | Model consent form and other related documentation given to participants and authorised surrogates | N/A |
| Biological specimens | 33 | Plans for collection, laboratory evaluation, and storage of biological specimens for genetic or molecular analysis in the current trial and for future use in ancillary studies, if applicable | N/A |

*It is strongly recommended that this checklist be read in conjunction with the SPIRIT 2013 Explanation & Elaboration for important clarification on the items. Amendments to the protocol should be tracked and dated. The SPIRIT checklist is copyrighted by the SPIRIT Group under the Creative Commons “[Attribution-NonCommercial-NoDerivs 3.0 Unported](http://www.creativecommons.org/licenses/by-nc-nd/3.0/)” license.
